# Stroboscopic training effects on athletic performance and cognitive function across populations, purposes, and skill types: a systematic review and meta-analysis of randomized controlled trials

**DOI:** 10.3389/fspor.2025.1705693

**Published:** 2025-12-11

**Authors:** Jintao Guo, Longtao Zhao, Guangsong Liu, Haobai Li, Jianzhong Wu

**Affiliations:** 1 College of Competitive Sports, Beijing Sport University, Beijing, China; 2 University of Jinan, Jinan, Shandong, China

**Keywords:** stroboscopic training, cognitive, athletic performance, meta-analysis, systematic review

## Abstract

**Background:**

This systematic review and meta-analysis aimed to evaluate the effects of stroboscopic training on cognitive function and athletic performance across different populations, purposes, and skill types.

**Methods:**

A comprehensive search of PubMed, Web of Science, SPORTDiscus, and Cochrane Library databases was conducted through August 2025. Only randomized controlled trials (RCTs) published in English that examined the effects of stroboscopic training on cognitive function (e.g., attention, perceptual ability, information processing speed) and athletic performance were included. Fourteen RCTs met the inclusion criteria, with 8 evaluating cognitive outcomes and 8 evaluating athletic performance.

**Results:**

Meta-analysis revealed that stroboscopic training produced a moderate positive effect on overall cognitive function (SMD = 0.64, 95% CI: 0.29 to 0.98, *p* < 0.01, *I*^2^ = 81%) and a moderate effect on athletic performance (SMD = 0.58, 95% CI: 0.37 to 0.78, *p* < 0.01, *I*^2^ = 64%). Total intervention duration was significantly positively correlated with effect size (cognitive: *b* = 0.0016, *p* < 0.01; athletic: *b* = 0.001, *p* < 0.01).

**Conclusions:**

These findings suggest that stroboscopic training, particularly protocols lasting 6–10 weeks with 2–3 sessions per week of 10–20 min each, can effectively enhance cognitive function and athletic performance, providing a promising neurocognitive training approach for sport training and clinical rehabilitation. This review followed PRISMA guidelines and was registered with PROSPERO (CRD420251070243).

**Systematic Review Registration:**

identifier, CRD420251070243.

## 1 Introduction

Optimizing perceptual–motor performance through innovative training methods has become increasingly important in sports science and rehabilitation as practitioners seek evidence-based strategies to enhance functional outcomes. Stroboscopic training, which uses liquid crystal glasses that alternate between transparent and opaque states, has emerged as a promising approach for inducing controlled visual perturbation during physical activity. These intermittent viewing conditions disrupt the continuity of visual feedback, requiring individuals to adopt alternative sensory strategies, heightened attentional control, and increased reliance on predictive mechanisms. Modeling-based interpretations further suggest that such constrained visual environments introduce functional instability into perceptual–motor systems, thereby promoting adaptive learning under ecologically relevant conditions (1). However, the mechanisms underlying these strategies and processes remain incompletely understood and warrant systematic investigation.

**Table 1:** Characteristics of included studies.

Study	Sample size	Age	Sex men/women	Intervention	Duration	Frequency	Session number	Session duration	Outcome measures
Choi et al. (2024) (15)	SV (20)	24.10 ± 3.84	10/10	SV + PFT	8 weeks	3times/week	24	30 min	Cognitive Abilities: FAAM-S↑; FAAM-ADL↑; IdFAI↑;I nversion error↑; Eversion error↑; Dorsiflexion error↑; Plantar flexion error↑; Giving-way episodes↑/Single-limb hopping test (s)↑; Figure-of-eight hop test (s)↑; Side-hop test (s)↑; Single-limb hurdle test (s)↑; Square hop test (s)↑; Single hop test (m)→/Visual Ability: COP-v (mm/s)↑
	CG (20)		9/11	PFT alone					
Fortes et al. 2023 (23)	SV (14)	25.2 ± 4.7	7/7	SV + small football competition	8 weeks	3times/week	24	20 min	Cognitive: MOT Threshold Speed→; MOT Score→; Decision-making Score→; →; Decision-making Pupil Diameter→; Decision-making Fixations Number→/Visual Ability:Anticipation Accuracy↑; Anticipation Response Time→; Anticipation Pupil Diameter→; Anticipation Fixations Number→; Anticipation Fixations Duration→; Decision-making Fixations Duration→
	CG (14)		7/7	small football competition					
Kim et al. 2021 (26)	SV (24)	27.17	17/7	SV + Conventional balance training	6 weeks	3 times/week	18	20 min	athletic performance: DFROM↑; SEBT-Ant↑; SEBT-PM↑; SEBT-PL↑; FAAM-ADL↑; FAAM-Sport↑
	CG (25)		12/13	Conventional balance training					
Argilés et al. 2025 (22)	SV (10)	32.05	5/5	stroboskopic vision	1 h	1 times/24–48 h	2	30 min	cognitive abilities: Dynamic Visual Acuity–45°/s, 100% →; Dynamic Visual Acuity–45°/s, 10%→; Dynamic Visual Acuity–26.5°/s, 100%→; Dynamic Visual Acuity–26.5°/s, 10%→; Multiple Object Tracking (MOT) NR
	CG (10)		5/5	Passive control					
Lee et al. 2024 (17)	SV (25)	21.5	13/12	SV + balance training	4 weeks	3 times/week	12	20 min	cognitive abilities: FAAM-Activities of Daily Living→; FAAM-Sport→; Ankle Instability Instrument→
	CG (25)		12/13	balance training					
Lee et al. 2021 (16)	SV (14)	21.5	6/8	SV + Dynamic balance training	4 weeks	3 times/week	12	20 min	Visual Ability: DPSI-SV↑; Area-SV→: Area-EC→/athletic performance: DPSI-EO↑; Area-EO→; FAAM ADL→; FAAM Sport→
	CG (14)		8/6	Dynamic balance training					
Li et al. 2024 (24)	SV (10)	21.7 ± 1.3	10/0	SV + Real-time feedback system	4 weeks	3 times/week	12	10 min	cognitive abilities: Duration judgment error↑/athletic performance: Speed control error→: Curling scoring↑
	CG (10)		10/0	Real-time feedback system					
Ellison et al. 2020 (18)	SV (31)	21.08	31/0	SV + Standard visual training	N/A	1	1	7–8 min	Visual Ability: VS accuracy 10 days→; VS speed 10 days→/athletic performance:EHC Immediately post↑; EHC 10 min post↑; EHC 10 days post↑
	CG (31)		31/0	Standard visual training					
Uzlasir et al. 2021 (11)	SV (13)	19.77	7/6	SV + Supervised balance training	6 weeks	3 times/week	18	15–20 min	cognitive abilities: Cz Theta↑; Cz Alpha↑; Cz Beta LF→; Cz Beta HF→/Visual Ability: Occipital Theta→; Occipital Alpha→; Occipital Beta LF→; Occipital Beta HF→
	CG (13)		7/6	Supervised balance training					
Zwierko et al. 2023 (9)	SV (25)	16.5 ± 0.6	13/12	SV + Volleyball-specific training	6 weeks	3 times/week	18	15 min	cognitive abilities: Complex Reaction Speed↑/athletic performance: Reactive Agility↑; Simple Motor Time↑; Simple Reaction Time→/Visual Ability: Saccade Dynamics↑; Sensory Sensitivity→
	CG (25)		13/12	Volleyball-specific training					
Zwierko et al. 2024a (10)	SV (25)	16.5 ± 0.6	13/12	SV + Volleyball-specific training	6 weeks	3 times/week	18	45.0 ± 1.4 min	cognitive abilities: Volleyball-specific blocking reaction speed→/athletic performance: Explosive leg strength→
	CG (25)		13/12	Volleyball-specific training				46.1 ± 2.0 min	
Zwierko et al. 2024b (25)	SV (25)	16.5 ± 0.6	13/12	SV + Volleyball-specific training	6 weeks	3 times/week	18	25–30 min	athletic performance: REAC-INDEX↑; CODS→; RA→
	CG (25)		13/12	Volleyball-specific training					
Zwierko et al. 2024c (30)	SV (22)	23.6 ± 4.4	22/0	SV + Ball-specific	Single session	2	2	20 min	athletic performance: RA without ball→; RA with ball→
	CG (22)		22/0	Ball-specific					
Palmer et al. 2022 (27)	SV (22)	11.2 ± 1.3	18	SV + Football training	4 weeks	1 times/week	4	20 min	athletic performance:Dribbling time →; Number of touches ↑
	CG (22)		18	Football training					

Despite growing interest in stroboscopic training, the neurophysiological and behavioral mechanisms responsible for its effects remain only partially understood. Existing studies indicate that intermittent visual occlusion alters reliance on visuospatial memory, predictive control, and distributed neural networks, yet the extent to which these adaptations translate into robust performance enhancements is inconsistent across studies (1). Although neurophysiological evidence has demonstrated changes in cortical activation patterns and prolonged response latencies under stroboscopic conditions (2), findings regarding motor accuracy, visuomotor timing, and cognitive responsiveness vary widely across task types and methodological designs. These inconsistencies highlight unresolved questions concerning the temporal thresholds, task-specific demands, and network-level processes required to sustain performance under intermittent visual constraints.

Sensory reweighting provides a useful conceptual framework for explaining how individuals adapt to stroboscopic perturbation by dynamically adjusting reliance on visual, vestibular, and proprioceptive inputs (3). Evidence shows that binocular integration offers advantages over monocular processing under intermittent viewing conditions (4), and that performance remains stable only when visual sampling intervals fall within specific temporal limits (4, 5). Importantly, intermittent visual disruption differs from complete visual elimination by preserving partial perceptual access while still promoting skill acquisition (6). Additional findings demonstrate improvements in motion sensitivity, transient attention, vigilance maintenance, anticipatory timing, and modulations in cortical oscillations associated with attention and sensorimotor integration (7–11). Meta-analytic and systematic reviews have further revealed moderate to large effects on time- and accuracy-related outcomes among athletes (13–17). Nonetheless, discrepancies remain, including selective enhancement of central but not peripheral motion sensitivity (18) and heterogeneous responses among individuals with chronic ankle instability (19, 20). Collectively, these findings underscore the need for a comprehensive synthesis of the existing evidence.

Given these inconsistencies, the present systematic review and meta-analysis aims to comprehensively evaluate the effects of stroboscopic training on athletic performance and cognitive function across different populations, training purposes, and skill types. Specifically, we seek to: (1) classify and examine methodological characteristics across existing studies; (2) quantify the magnitude of stroboscopic training effects on athletic, perceptual, and cognitive outcomes; and (3) identify moderators such as population characteristics, task domain, skill complexity, and training parameters. Based on prior empirical evidence, we hypothesize that stroboscopic training will yield small-to-moderate positive effects across outcome domains, with potentially larger benefits in tasks involving predictive control, visual motion sensitivity, or higher-order perceptual–motor integration. Through this synthesis, we aim to clarify the robustness and generalizability of stroboscopic training effects and provide evidence-based recommendations for future practice and research.

## 2 Materials and methods

This systematic review and meta-analysis was conducted following the Preferred Reporting Items for Systematic Reviews and Meta-Analyses (PRISMA) guidelines and was registered in PROSPERO (registration number: CRD420251070243), an international prospective register of systematic reviews.

### 2.1 Literature search

PubMed, Web of Science, SPORTDiscus, and Cochrane Library databases were searched by two authors (JG and ZL) in August 2025. The search strategy was developed *a priori* in accordance with PRISMA guidelines, and Boolean operators (AND, OR) were used to combine predefined keyword groups. The primary search terms included stroboscopic-related terminology (stroboscopic, strobe, “stroboscopic glasses”, “stroboscopic goggles”, “stroboscopic training”, “stroboscopic vision”, “visual occlusion”, “intermittent vision”, “liquid crystal glasses”, “visual disruption”), exercise-related terminology (training, exercise, intervention, therapy), and outcome-related terminology (athletic, sport, performance, cognitive, “motor control”, balance, agility, “reaction time”).

In this review, athletic performance is conceptualized as a multidimensional construct encompassing: (1) fundamental motor abilities (reaction time, balance, motor control); (2) sport-specific technical skills (dribbling performance, blocking speed, shooting accuracy); and (3) complex motor performance (change-of-direction speed, functional movement tests). This classification acknowledges the hierarchical and domain-specific nature of athletic performance, ranging from basic motor capacities to integrated sport-specific competencies. while cognitive outcomes include attention, visual sensitivity, processing speed, and anticipatory timing.

In addition to database searches, the reference lists of all included articles and relevant systematic reviews were manually screened to ensure completeness. The search was restricted to English-language publications. Any disagreements during the screening process were resolved through discussion between the two authors (JG and ZL). The full search strategy, including Boolean combinations and synonym groups, is provided in Supplementary Table S1.

### 2.2 Search strategy

All included studies must be published articles. Inclusion criteria were based on the PICOS framework: (1) evaluated the effects of stroboscopic training on athletic performance and/or cognitive function, including healthy individuals, athletes, or patient populations with specific conditions; (2) employed randomized controlled trial (RCT) or randomized crossover trial designs; (3) stroboscopic training used as a standalone intervention or combined with other therapeutic approaches, with control groups receiving only the other intervention components when used in combination; (4) included active control groups (receiving other visual training interventions) or non-active control groups (no intervention, placebo, or usual care); (5) reported primary quantitative data evaluating athletic performance and/or cognitive function, with adequate statistical parameters (means, standard deviations, sample sizes) for meta-analysis; (6) peer-reviewed published articles. Articles were excluded if they were: (1) review articles; (2) systematic reviews and meta-analyses; (3) conference abstracts; (4) conference papers; (5) articles without specific research outcome data; (6) duplicate publications; (7) non-randomized controlled trial designs; (8) observational studies, case reports, or case series; (9) studies lacking statistical parameters necessary for meta-analysis; (10) non-English publications.

### 2.3 Data extraction

The primary data for this study were outcome measures of athletic performance and cognitive function-related indicators assessed in the included studies. Other relevant extracted data included study participant characteristics (sample size, age, sport type, training years, physical condition), intervention details (intervention purpose, training duration, training frequency, session length, total number of training sessions), outcome assessment methods (cognitive ability tests, athletic performance tests). If the data is missing, contact the corresponding author. If no response is received within 48 h, the study is excluded.

### 2.4 Quality assessment

The quality of included studies was independently assessed by two authors (GT and HB) according to the Physiotherapy Evidence Database (PEDro) principles. The PEDro scale was specifically designed to evaluate the methodological quality of randomized controlled trials of physical interventions, making it particularly suitable for assessing the quality of stroboscopic training intervention studies in this research. This scale evaluates key factors such as randomization, blinding, and allocation concealment, which are crucial for ensuring the internal validity of studies included in this systematic review and meta-analysis. Specifically, the PEDro scale consists of 11 items, for which we were required to respond with “no” or “yes”. For each “no” or “yes” response, we assigned a value of 0 or 1, respectively. The total score for each study ranged from 0 to 11. Since blinding (particularly of participants and therapists) is not easily implemented in sport training intervention trials—participants can clearly perceive the visual occlusion effects of stroboscopic glasses—we adjusted the methodological quality classification for each article, considering the eligibility criteria as previously described [total score: ≥6 (“high quality, low risk of bias”); score: 4–5 (“acceptable quality, moderate risk of bias”); score: ≤3 (“low quality, high risk of bias”)].

### 2.5 Statistical analysis

Data extraction and statistical analyses were performed independently by two researchers. The primary effect size measure was the standard-ized mean difference (Cohen's d) with 95% confidence intervals (CI) to evaluate the effects of stroboscopic training on athletic performance and cognitive function.

A random-effects model (DerSimonian-Laird method) was employed for meta-analysis to account for between-study heterogeneity. Heterogeneity was assessed using the *I*^2^ statistic, where *I*^2^ < 25% indicated low heterogeneity, 25%–50% indicated moderate heterogeneity, and >50% indicated substantial heterogeneity. When *I*^2^ > 50%, sources of heterogeneity were explored through subgroup and sensitivity analyses.

Subgroup analyses were stratified by the following variables: participant age (adolescent vs. adult), physical condition (CAI patients vs. healthy individuals), study context (rehabilitation vs. sport performance), athletic status (athletes vs. non-athletes), training experience (<5 years vs. >5 years), and skill type (fine vs. gross motor skills). Additionally, separate meta-analyses were conducted for specific cognitive dimensions (attention, perceptual ability, and information processing speed).

Meta-regression analysis was performed to explore the dose-response relationship between total intervention duration and effect size. Sensitivity analysis using the leave-one-out method assessed the influence of individual studies on the pooled effect estimate. Publication bias was evaluated through funnel plots, Egger's test (*t*-test), and Begg's test. When bias was found, the bias-free correction estimation was performed by the cut-and-fill method. All statistical analyses were conducted using R software (version 4.3.0).

## 3 Results

### 3.1 Study selection

**Figure 1:**
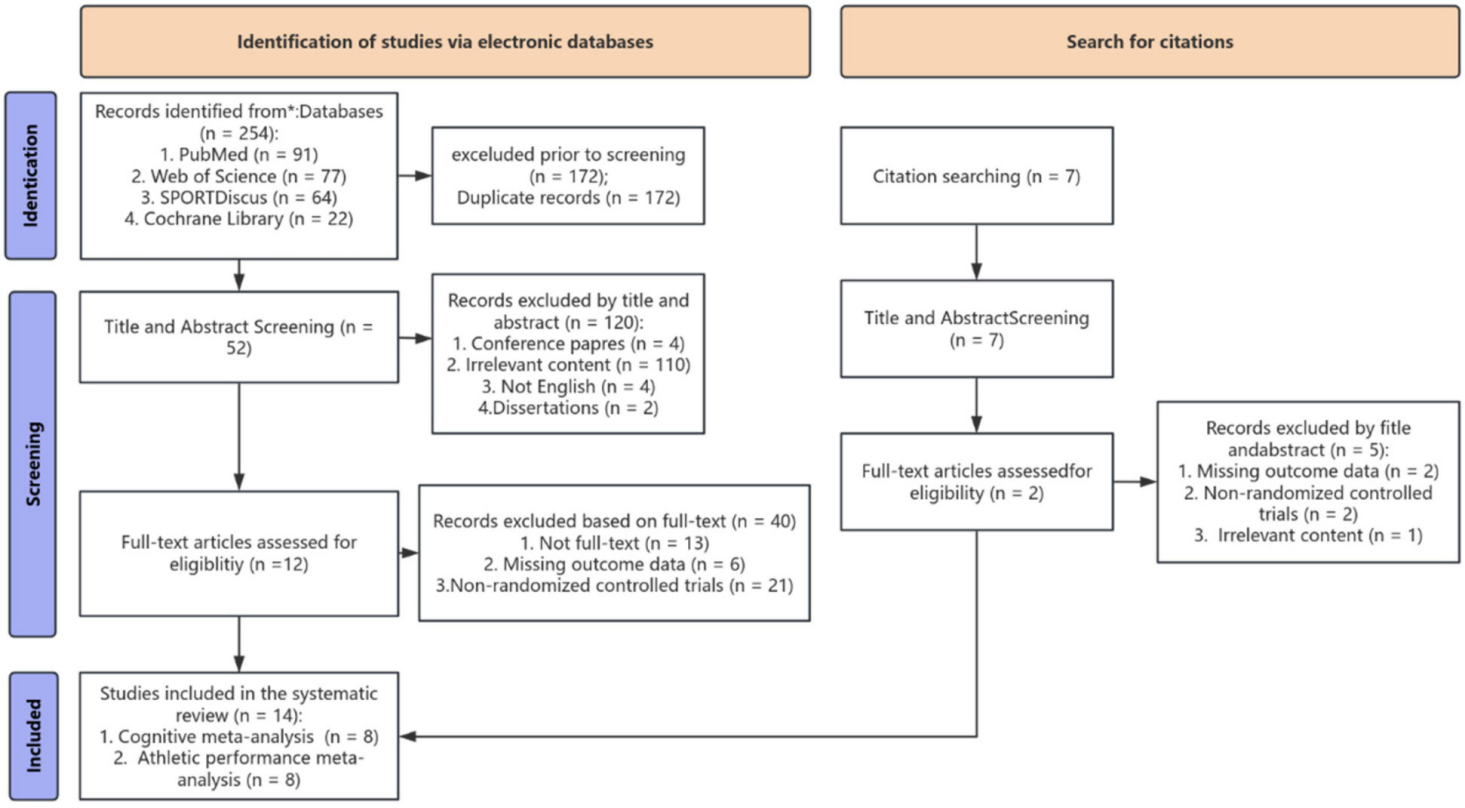
PRISMA.

Figure 1 illustrates the study selection flowchart. Database searches yielded 254 records (PubMed: 91; Web of Science: 77; SPORTDiscus: 64; Cochrane Library: 22). Following duplicate removal (*n* = 82), 172 records underwent screening. Title and abstract screening excluded 120 records: 110 for irrelevance, 4 conference papers, 4 non-English publications, and 2 dissertations. Full-text assessment excluded an additional 40 articles: 21 non-RCTs, 13 with unavailable full text, and 6 lacking outcome measures. Citation searching identified 2 additional eligible studies. Fourteen studies were ultimately included for meta-analysis [8 for cognitive outcomes (9, 10, 15, 18, 22–25); 8 for athletic performance (10, 15–17, 24–27)].

### 3.2 Characteristics of included studies

Among the 14 included studies, five articles implemented stroboscopic training combined with balance interventions, utilizing functional balance training (Table 1) (15), conventional balance training (26), dynamic balance training (16, 17), or supervised balance protocols (11) as primary interventions. Three studies focused on volleyball athletes and employed sport-specific volleyball training combined with stroboscopic stimulation (9, 10, 25). Two studies addressed football/soccer performance using small-sided games (23) or standard football training (27) under stroboscopic conditions. The remaining studies utilized diverse modalities including ball-specific training (25), standard visual training (18), real-time feedback systems (24), and pure stroboscopic visual stimulation (22) to enhance perceptual-motor performance.

Across the 14 included RCTs, a diverse array of outcome measures were assessed to evaluate the effects of stroboscopic training. The most frequently measured indicators were time-based performance metrics (reported in 10 studies), including reaction time, movement execution time, and reactive agility measured in seconds. Accuracy-based outcomes were assessed in 7 studies, encompassing anticipation accuracy, technical skill success rates, and ball control precision measured as percentages. Several studies also examined cognitive abilities such as FAAM scores, dynamic visual acuity, and multiple object tracking performance. Visual-specific measures including center of pressure velocity (COP-v), dynamic postural stability index (DPSI), and Star Excursion Balance Test (SEBT) scores were reported in 6 studies. Additionally, neurophysiological markers such as theta and alpha wave activity were measured in one study, providing insights into the neural adaptations to stroboscopic training.

The included studies varied in intervention duration, ranging from single-session acute interventions lasting one hour (22) to extended protocols spanning 8 weeks (15, 23). The majority of studies implemented interventions lasting 4–6 weeks (9 studies), with training frequencies predominantly set at 3 sessions per week (9 studies) and individual session durations typically ranging from 20 to 30 min. Sample sizes ranged from 10 to 50 participants per study, with participant ages spanning from 11.2 to 32 years, though most studies focused on adolescent and young adult populations aged 16–25 years. Training experience among participants varied across studies, with volleyball players having 6.65 ± 2.24 years of sport-specific practice (9, 10, 25, 30).

### 3.3 Quality assessment of included studies

This systematic review used the PEDro scale to assess the risk of bias for all included studies regarding cognitive function and athletic performance outcomes. This assessment method, specifically designed for the methodological quality of physical intervention trials, may yield different results compared to other assessment tools. Specifically, 21.4% (3/14) of studies achieved the highest quality standard (PEDro score of 9), 14.3% (2/14) scored 8 points, and 64.3% (9/14) scored 7 points. Although all studies met the high-quality threshold (≥6 points), there remains room for improvement in specific methodological areas: only 14.3% (2/14) of studies implemented allocation concealment, 7.1% (1/14) achieved investigator blinding, and 35.7% (5/14) achieved assessor blinding. Notably, due to the inherent nature of stroboscopic training, no studies were able to achieve participant blinding, which represents an inherent limitation of this type of intervention research.

### 3.4 Cognitive meta-analysis

#### 3.4.1 Effects of stroboscopic training on cognitive function

**Figure 2:**
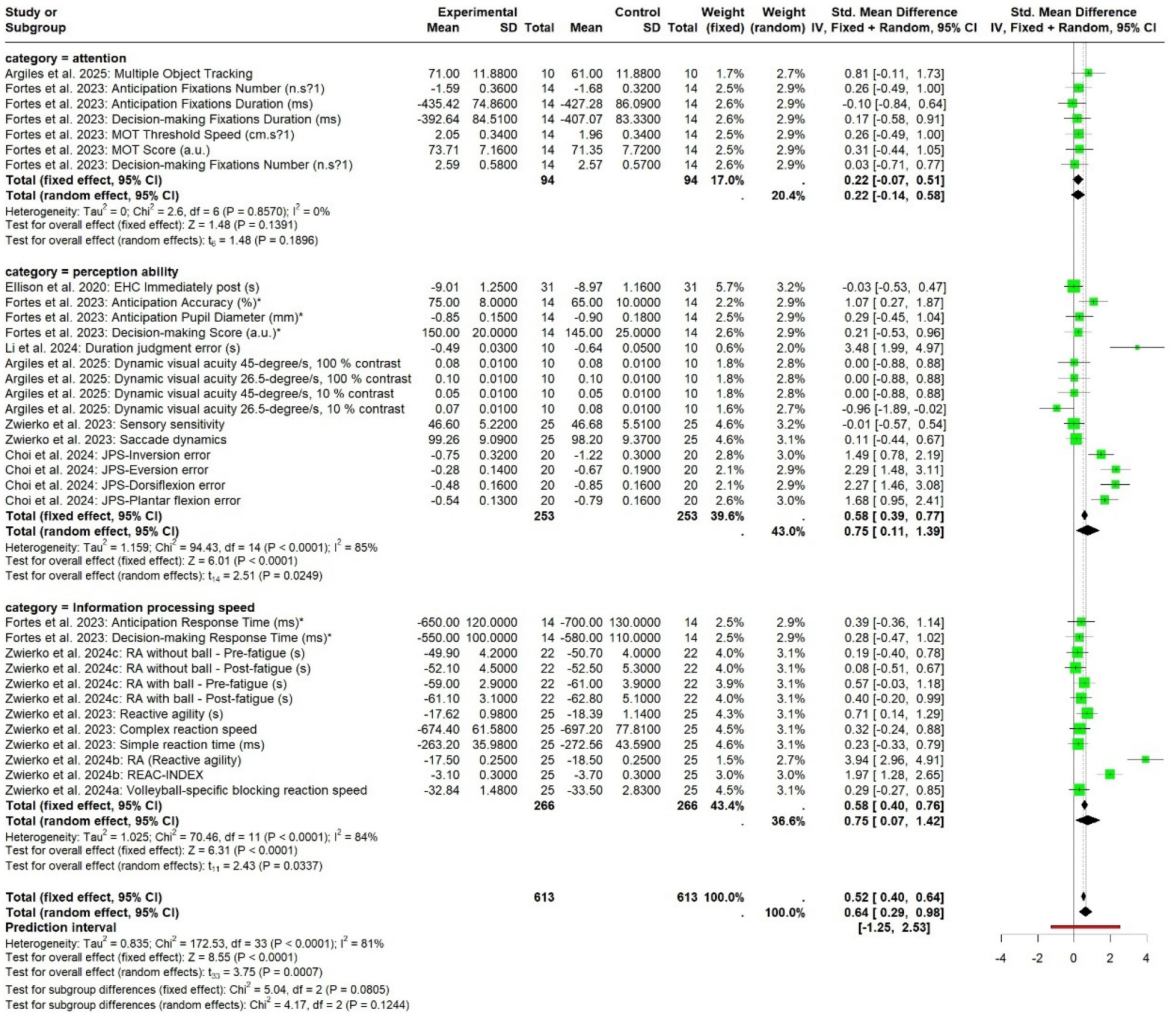
The pooled effect size of SV on cognitive function levels.

**Figure 3:**
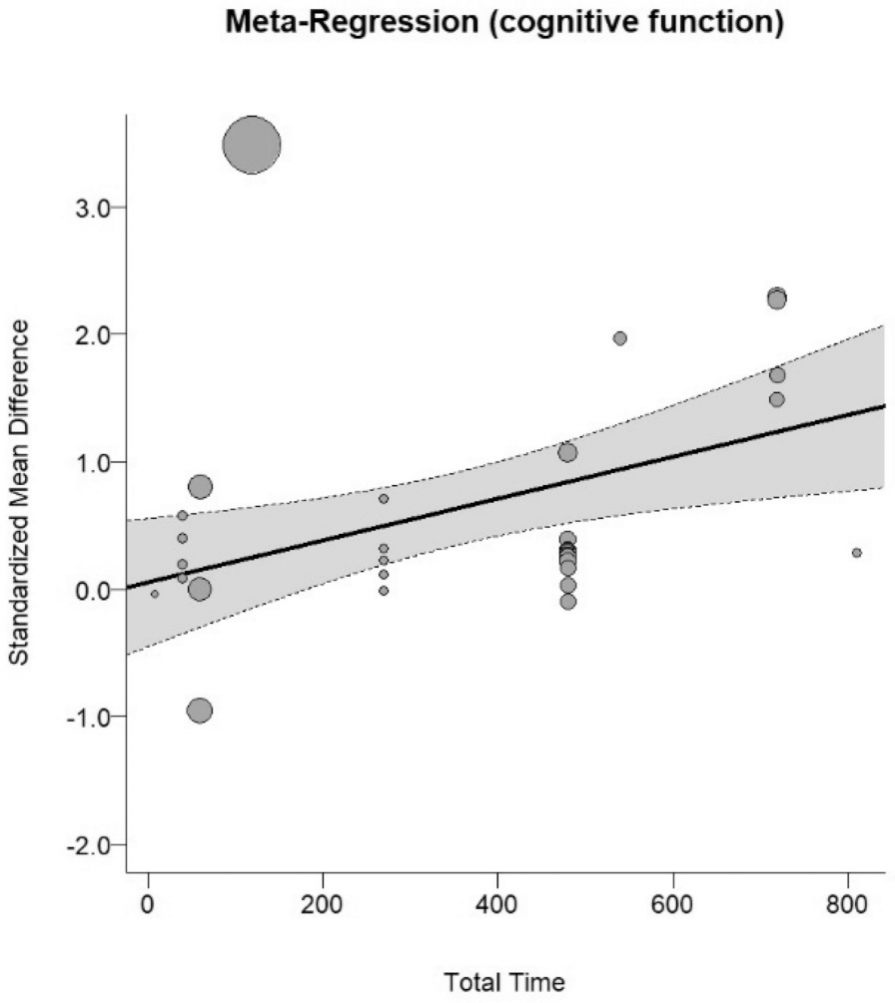
Dose-response curve on cognitive function.

**Figure 4:**
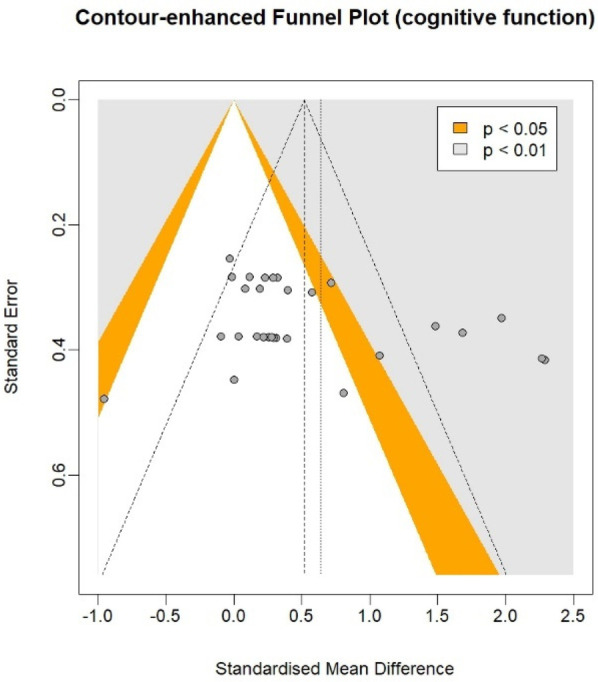
Funnel plot on cognitive function.

In the cognitive meta-analysis, stroboscopic training demonstrated a significant moderate positive effect on overall cognitive function (SMD = 0.64; 95% CI: 0.29 to 0.98; PI: −1.25 to 2.53; *p* < 0.01; Figure 2), with substantial heterogeneity (*I*^2^ = 81%; *p* < 0.01). Regression analysis examining the relationship between total intervention duration and effect size revealed a significant positive correlation (*b* = 0.0016; *p* < 0.01; Figure 3), indicating that longer intervention durations yielded larger effect sizes. The funnel plot (Figure 4) and Egger's test (*t* = 2.62, *p* = 0.01) indicated a potential risk of publication bias, but the Trim and Fill method for sensitivity analysis showed that the pooled effect size (SMD = 0.34, *p* < 0.01) was robust.

Separate meta-analyses were conducted for test measures involving attention, perceptual ability, and information processing speed. Results showed that stroboscopic training produced a small, non-significant effect on attention (SMD = 0.22; 95% CI: −0.07 to 0.51; *p* = 0.14), with minimal heterogeneity (*I*^2^ = 0%; *p* = 0.86). Stroboscopic training yielded a large and statistically significant effect on perceptual ability (SMD = 0.75; 95% CI: 0.11 to 1.39; *p* = 0.02), though with substantial heterogeneity (*I*^2^ = 85%; *p* < 0.001). For information processing speed, stroboscopic training produced a large and statistically significant effect (SMD = 0.75; 95% CI: 0.07 to 1.42; *p* < 0.01), also with substantial heterogeneity (*I*^2^ = 84%; *p* < 0.001).

#### 3.4.2 Sensitivity and subgroup analyses

Sensitivity analyses confirmed the robustness of previous findings for stroboscopic training effects on attention [SMD = 0.22; *P* = 0.14; Supplementary Figure SA], perceptual ability [SMD = 0.75; *P* = 0.02; Supplementary Figure SB], and information processing speed [SMD = 0.75; *P* = 0.03; Supplementary Figure SC].

We conducted subgroup analyses for significant cognitive abilities (perceptual ability and information processing speed) to identify potential moderating factors. Subgroup analysis for perceptual ability revealed that effects were not moderated by participant age group or skill classification, but training experience significantly influenced the intervention effects of stroboscopic training (Table 2). Subgroup analysis for information processing speed showed that effects were not moderated by participant age group or training experience (Table 2).

**Table 2:** The subgroup analysis results of perceptual ability and information processing speed.

Sub-group	N (K)	SMD	95% CI	P	*T* ^2^	*I* ^2^	Sig. Mod.
Perception ability (*N* = 6)							
Age grades							
Juvenile	1 (2)	0.05	NA	NA	NA	NA	ns
Adult	5 (13)	0.87	0.13; 1.6	0.02	1.29	86	ref
Years of training							
No training	1 (4)	−0.22	NA	NA	NA	NA	sig**
<5 years	2 (5)	1.98	1.62; 2.35	<0.01	0.24	46	ref
>5 years	3 (6)	0.18	−0.08; 0.58	0.17	0.03	18	sig**
Skill Classification							
Fine	2 (5)	0.43	−1.62; 2.48	0.59	2.46	84	ns
Gross	4 (10)	0.91	0.24; 1.58	0.01	0.75	86	ref
Information processing speed (*N* = 5)							
Age grades							
Juvenile	2 (5)	1.4	0.07; 2.73	0.04	2.19	93	ns
Adult	3 (7)	0.31	0.07; 0.54	0.01	0	0	ref
Years of training							
<5 years	1 (4)	0.31	0.01; 0.6	0.04	0	0	ref
>5 years	4 (8)	0.98	−0.09; 2.06	0.07	1.52	89	ns

**Significant.

### 3.5 Athletic performance meta-analysis

#### 3.5.1 Effects of stroboscopic training on athletic performance outcomes

**Figure 5:**
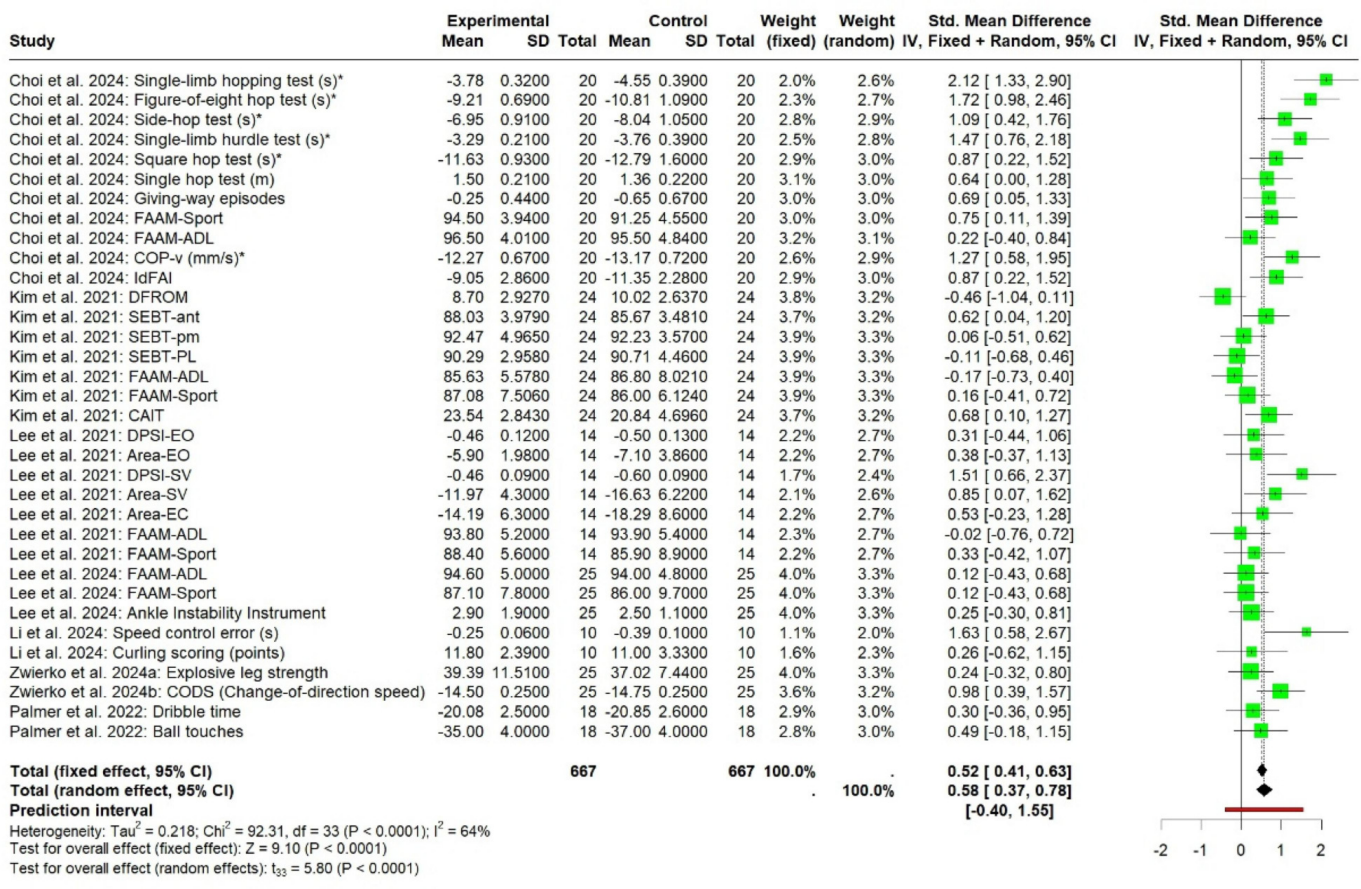
The pooled effect size of SV on athletic performance meta-analysis levels.

**Figure 6:**
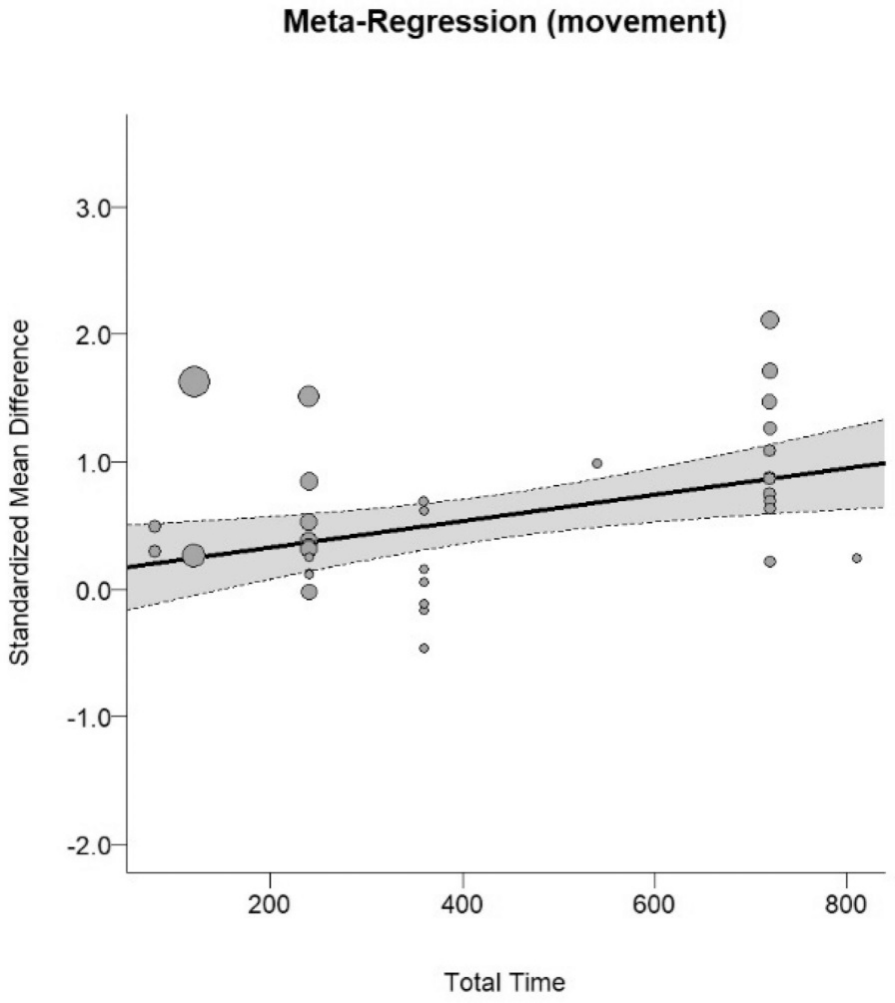
Dose-response curve of athletic performance.

**Figure 7:**
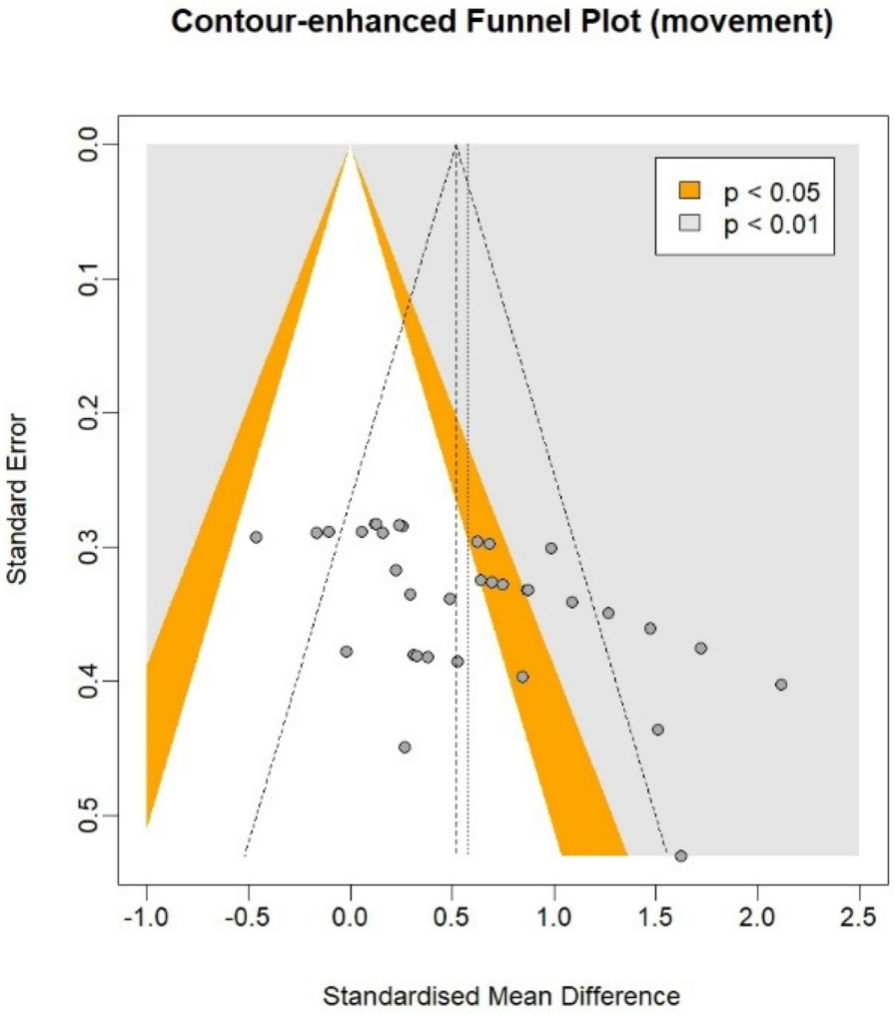
Funnel plot of athletic performance.

In the athletic performance meta-analysis, stroboscopic training demonstrated a significant moderate positive effect on overall athletic performance outcomes (SMD = 0.58; 95% CI: 0.37 to 0.78; PI: −0.4 to 1.55; *p* < 0.01; Figure 5), with substantial heterogeneity (*I*^2^ = 64%; *p* < 0.01). Regression analysis examining the relationship between total intervention duration and effect size revealed a significant positive correlation (*b* = 0.001; *p* < 0.01; Figure 6), indicating that longer intervention durations yielded larger effect sizes. The funnel plot (Figure 7) and Egger's test (*t* = 3.84, *p* < 0.01) indicated a potential risk of publication bias, but the Trim and Fill method for sensitivity analysis showed that the pooled effect size (SMD = 0.39, *p* < 0.01) was robust.

#### 3.5.2 Sensitivity and subgroup analyses

Sensitivity analysis confirmed the robustness of stroboscopic training effects on athletic performance outcomes [SMD = 0.58; *P* < 0.01; Supplementary Figure SD]. Subgroup analyses for athletic performance meta-analysis revealed that the effectiveness of stroboscopic training was not moderated by participant age group, physical condition, or study context. However, stroboscopic training produced significantly greater positive effects in athletes compared to non-athletes (Table 3).

**Table 3:** The subgroup analysis results of athletic performance.

Sub-group	N (K)	SMD	95% CI	P	*T* ^2^	*I* ^2^	Sig. Mod.
Age grades							
Juvenile	2 (4)	0.51	0.4; 0.64	<0.01	0.03	21	ns
Adult	4 (30)	0.59	0.37; 0.8	<0.01	0.24	67.3	ref
Population							
Chronic Ankle Instability(CAI)	3 (28)	0.57	0.34; 0.81	<0.01	0.25	68	ref
Healthy individuals	3 (6)	0.59	0.08; 1.1	<0.01	0.11	40	ns
Context							
Rehabilitation	3 (28)	0.57	0.34; 0.81	<0.01	0.25	68	ref
sport	3 (6)	0.59	0.08; 1.1	<0.01	0.11	40	ns
Exercise situation							
athletes	4 (17)	0.89	0.6; 1.17	<0.01	0.18	57	ref
Non-athlete	2 (17)	0.23	0.08; 0.39	<0.01	0.07	38	sig***

***Very significant.

## 4 Discussion

This systematic review and meta-analysis evaluated how stroboscopic training affects cognitive function and athletic performance across different populations, purposes, and skill types. Analyzing fourteen randomized controlled trials, we found that stroboscopic training produced moderate positive effects on overall cognitive function (SMD = 0.64, 95% CI: 0.29 to 0.98, *p* < 0.01) and athletic performance (SMD = 0.58, 95% CI: 0.37 to 0.78, *p* < 0.01). These results partially align with our hypothesis of small-to-moderate effects across outcome domains. Interestingly, we observed substantial benefits for perceptual ability (SMD = 0.75) and information processing speed (SMD = 0.75), supporting our prediction about enhanced effects in domains requiring predictive control and perceptual-motor integration. However, we found no significant effects on attention (SMD = 0.22, *p* = 0.14), indicating more selective enhancement than we initially expected.

### 4.1 Methodological quality and publication bias

Quality assessment showed all included studies met high-quality standards (PEDro score ≥6), with 21.4% achieving the highest rating (score of 9). Yet several methodological limitations deserve attention. Only 14.3% of studies implemented allocation concealment, 7.1% achieved investigator blinding, and 35.7% achieved assessor blinding. Most importantly, no studies could blind participants because athletes clearly perceive the visual occlusion effects of stroboscopic glasses—an unavoidable limitation that may introduce expectancy effects (1, 21).

Publication bias analysis revealed potential selective reporting through funnel plot asymmetry and Egger's test for both cognitive (*t* = 2.62, *p* = 0.01) and athletic performance outcomes (*t* = 3.84, *p* < 0.01). Nevertheless, Trim and Fill sensitivity analyses showed our pooled effect sizes remained statistically significant after correction (cognitive: SMD = 0.34, *p* < 0.01; athletic: SMD = 0.39, *p* < 0.01), confirming the robustness of our core findings. The substantial heterogeneity we observed (cognitive: *I*^2^ = 81%; athletic: *I*^2^ = 64%) reflects variations in training protocols, outcome measures, and participant characteristics, which we systematically addressed through subgroup and meta-regression analyses.

These methodological considerations highlight the need for future studies to implement rigorous blinding procedures for assessors and statisticians, pre-register protocols to minimize selective reporting, and adopt standardized outcome measures to reduce heterogeneity (12).

### 4.2 Visuomotor mechanisms supporting performance gains

The selective enhancement of perceptual ability and information processing speed—while attention remains unaffected—suggests stroboscopic training works through specific visuomotor integration pathways rather than broad cognitive enhancement. This pattern strongly aligns with sensory reweighting theory (3, 36), which proposes that intermittent visual occlusion forces dynamic adjustment of reliance on visual, vestibular, and proprioceptive inputs. Our findings extend this framework by demonstrating that such adaptations translate into measurable improvements in visuomotor timing and motion sensitivity.

Neurophysiological studies support these behavioral findings. Stroboscopic conditions prolong visual motion perception through altered central nervous system processing (2) and promote phase synchronization within brain regions involved in sensory processing and balance control (28). The specificity of our results—enhanced perceptual abilities without generalized attentional improvements—indicates that training under intermittent viewing conditions primarily strengthens visual-motor pathways involved in predictive control and anticipatory timing (7, 9, 10). Importantly, intermittent visual disruption differs fundamentally from complete visual elimination by preserving partial perceptual access while still promoting skill acquisition (6), which may explain why stroboscopic training produces selective rather than global enhancements.

Research showing advantages of binocular integration over monocular processing under stroboscopic conditions (4) and stable performance within specific temporal sampling limits (4, 5) further supports the notion that these adaptations reflect optimized visuomotor coordination rather than compensatory strategies. Studies examining central vs. peripheral motion sensitivity reveal that stroboscopic training selectively enhances central motion processing while leaving peripheral sensitivity unchanged (18), providing additional evidence for pathway-specific neural plasticity. The dose-response relationships we identified (cognitive: *b* = 0.0016, *p* < 0.01; athletic: *b* = 0.001, *p* < 0.01) indicate progressive neuroplastic changes consistent with use-dependent plasticity principles, where repeated exposure to controlled visual perturbation strengthens neural circuits mediating perceptual-motor integration (11, 30).

### 4.3 Neurophysiological and skill-specific mechanisms

Beyond visuomotor pathways, several converging mechanisms explain the observed performance enhancements. At the neurophysiological level, stroboscopic training appears to induce adaptive plasticity in cortical networks, evidenced by modulations in theta and alpha oscillations associated with attention and sensorimotor integration (11, 12). These neural adaptations may help individuals develop tolerance to visual disruption, similar to desensitization processes in high-pressure athletic situations (8).

Our subgroup analyses revealed that athletic expertise significantly moderated training effects, with athletes showing larger benefits (SMD = 0.89) than non-athletes (SMD = 0.23, *p* < 0.01). This population-specific response pattern suggests a facilitation model, where stroboscopic training amplifies existing perceptual-motor capabilities rather than creating entirely new skills (31, 32). The finding that training experience significantly influenced perceptual ability outcomes supports this interpretation, as skilled performers possess more developed neural scaffolding upon which stroboscopic training can build (29, 35).

Task context appears to strongly influence stroboscopic training effectiveness, with sport-specific integration yielding superior outcomes compared to isolated visual training (13, 14, 23, 27). This contextual specificity likely reflects ecological validity principles, where training effects transfer most effectively when practice conditions closely match performance demands. Studies implementing volleyball-specific training (9, 10, 25) and football-specific protocols (23, 27) under stroboscopic conditions demonstrated pronounced improvements, indicating that intermittent visual disruption enhances skill acquisition when embedded within meaningful action contexts.

For rehabilitation applications, the moderate effects observed in chronic ankle instability populations (15–17, 19, 20, 26) suggest potential therapeutic value, though adapted protocols accounting for initial functional limitations may be necessary. Research on neuromechanics during landing-cutting tasks in individuals with chronic ankle instability demonstrates that visual disruption affects postural control and movement patterns (17), highlighting both the challenges and opportunities for therapeutic applications. The portable nature of stroboscopic glasses (7, 37) enhances practical accessibility across diverse settings, making this training modality feasible for both performance enhancement and clinical rehabilitation contexts. Emerging evidence also suggests potential applications in proprioceptive training, as demonstrated by effects on blindfolded walking performance (38), expanding the possible uses of stroboscopic training beyond traditional sport settings.

### 4.4 Limitations and future directions

Several limitations must be acknowledged. First, the inability to achieve participant blinding introduces potential placebo and expectancy effects that cannot be fully separated from genuine training adaptations. Future research should incorporate active control conditions providing alternative forms of visual perturbation, enabling more precise isolation of stroboscopic-specific mechanisms.

Second, the predominance of short-term interventions (4–8 weeks) limits conclusions about long-term retention and transfer of benefits to real-world performance. While single-session acute interventions have shown immediate effects (22), longitudinal studies tracking performance over extended periods and examining maintenance following training cessation are critically needed (33).

Third, our exclusive inclusion of English-language publications may introduce language bias, potentially excluding relevant findings from non-English literature. Fourth, limited representation of clinical populations beyond chronic ankle instability restricts conclusions about therapeutic applications, highlighting the need for research examining stroboscopic training effects in diverse rehabilitation contexts including neurological disorders, balance impairments, and age-related decline (14, 34).

Fifth, our exclusive focus on randomized controlled trials (RCTs), while ensuring high internal validity, meant that some potentially informative studies using other methodological approaches were excluded. For example, recent research by Vasile and Stănescu (39) reported positive effects of long-term stroboscopic training on climbing performance through a 20-session intervention over one calendar year. Although their study employed selective distribution rather than randomization—with climbers allocated based on geographic location—their findings suggest potential benefits of extended training protocols in sport-specific contexts. Similarly, research employing sports vision training using the Senaptec Sensory Station (40) demonstrated improvements in shooting performance, though this computerized station-based approach differs fundamentally from stroboscopic training with intermittent visual occlusion during physical activity. Future systematic reviews might consider synthesizing evidence across different study designs with appropriate methodological considerations and subgroup analyses distinguishing RCTs from well-controlled quasi-experimental studies.

Methodologically, future research should prioritize standardized outcome measures enabling cross-study comparisons, as the substantial heterogeneity we observed partly reflects diverse assessment approaches. Development of consensus guidelines for stroboscopic training protocols—including recommendations for occlusion frequency, session duration, and progression principles based on population characteristics—would advance systematic implementation.

Our meta-regression findings indicate that total intervention duration positively predicts effect magnitude, but optimal dosage parameters for specific populations and outcomes remain unclear. Systematic investigation of dose-response relationships, examining various combinations of session frequency, duration, and training period length, could inform evidence-based prescription. Additionally, research exploring individual difference variables—such as baseline perceptual-motor skill, learning capacity, and intrinsic motivation—may identify predictors of training responsiveness and sustained engagement (15, 24, 25). Understanding factors that promote adherence and predict long-term outcomes would enable personalized implementation strategies maximizing individual benefits.

### 4.5 Conclusions

This systematic review and meta-analysis provides robust evidence that stroboscopic training effectively enhances cognitive function and athletic performance through targeted improvements in perceptual ability and information processing speed. The moderate effect sizes observed (cognitive: SMD = 0.64; athletic: SMD = 0.58), combined with significant dose-response relationships and population-specific response patterns, establish stroboscopic training as a viable neurocognitive intervention applicable across sport and rehabilitation contexts.

The selective enhancement of visuomotor integration capacities, without generalized cognitive improvements, indicates that intermittent visual occlusion operates through specific neural pathways involving sensory reweighting and perceptual-motor plasticity. Practical implementation should consider population characteristics, with sport-specific integration recommended for athletes and adapted protocols for clinical populations.

Optimal training protocols appear to involve 6–10 weeks of intervention with 2–3 sessions per week lasting 10–20 min each, though individualization based on specific goals and participant characteristics remains important. Future research should address current limitations through longitudinal designs, standardized assessment protocols, diverse clinical populations, and systematic investigation of optimal dosage parameters. Development of evidence-based implementation guidelines through collaboration among sports organizations, rehabilitation centers, and research institutions would facilitate broader adoption and maximize the potential benefits of this promising training modality for competitive sports and clinical rehabilitation applications.

## 5 Conclusions

The results from this systematic review and meta-analysis demonstrate that stroboscopic training effectively enhances both cognitive function and athletic performance. For cognitive outcomes, stroboscopic training primarily improves perceptual ability and information processing speed while showing minimal effects on attention, indicating selective enhancement. For athletic performance, athletes gain greater benefits compared to non-athletes. Training duration positively correlates with effect magnitude, supporting the implementation of sustained training protocols. These findings establish stroboscopic training as an effective neurocognitive training method that can be tailored to different population needs. It is recommended that sports organizations, rehabilitation centers, and related institutions collaborate to develop standardized implementation guidelines, optimize training parameters, and integrate stroboscopic training into sport training and rehabilitation systems to fully realize its application value in competitive sports and clinical rehabilitation.

## Data Availability

The original contributions presented in the study are included in the article/Supplementary Material, further inquiries can be directed to the corresponding author.

## Appendix A

**Pubmed (91):** (stroboscopic training OR Stroboscopic vision OR Intermittent Visual Occlusion Training OR Stroboscopic Visual Training) AND (exercise OR Physical Training OR sport OR sport performance OR athletic)

**Web of science (77):** TS = ((stroboscopic training OR “stroboscopic vision” OR “intermittent visual occlusion training” OR “stroboscopic visual training”) AND (exercise OR “physical training” OR sport* OR “sport performance” OR athletic*))

**SPORTDiscus with Full Text (64)** (TI stroboscopic training OR AB stroboscopic training OR KW stroboscopic training OR SU stroboscopic training OR TI “stroboscopic vision” OR AB “stroboscopic vision” OR KW “stroboscopic vision” OR SU “stroboscopic vision” OR TI “intermittent visual occlusion training” OR AB “intermittent visual occlusion training” OR KW “intermittent visual occlusion training” OR SU “intermittent visual occlusion training” OR TI “stroboscopic visual training” OR AB “stroboscopic visual training” OR KW “stroboscopic visual training” OR SU “stroboscopic visual training”) AND (TI exercise OR AB exercise OR KW exercise OR SU exercise OR TI “physical training” OR AB “physical training” OR KW “physical training” OR SU “physical training” OR TI sport* OR AB sport* OR KW sport* OR SU sport* OR TI “sport performance” OR AB “sport performance” OR KW “sport performance” OR SU “sport performance” OR TI athletic* OR AB athletic* OR KW athletic* OR SU athletic*)

**Cochrane Library (**22): (stroboscopic training OR “stroboscopic vision” OR “intermittent visual occlusion training” OR “stroboscopic visual training”) AND (exercise OR “physical training” OR sport* OR “sport performance” OR athletic*)

## References

[1] Appelbaum LG Cain MS Schroeder JE Darling EF Mitroff SR. Stroboscopic visual training improves information encoding in short-term memory. Atten Percept Psychophys. (2012) 74:1681–91. 10.3758/s13414-012-0344-6 22810559

[2] Hülsdünker T Fontaine G Mierau A. Stroboscopic vision prolongs visual motion perception in the central nervous system. Scand Med Sci Sports. (2023) 33:47–54. 10.1111/sms.14239 36111383

[3] Assländer L Peterka RJ. Sensory reweighting dynamics in human postural control. J Neurophysiol. (2014) 111:1852–64. 10.1152/jn.00669.2013 24501263 PMC4044370

[4] Bennett S Ashford D Rioja N Coull J Elliott D. Integration of intermittent visual samples over time and between the eyes. J Mot Behav. (2006) 38:439–50. 10.3200/JMBR.38.6.439-450 17138528

[5] Elliott D Zuberec S Milgram P. The effects of periodic visual occlusion on ball catching. J Mot Behav. (1994) 26:113–22. 10.1080/00222895.1994.9941666 15753064

[6] Bennett SJ Hayes SJ Uji M. Stroboscopic vision when interacting with multiple moving objects: perturbation is not the same as elimination. Front Psychol. (2018) 9:1290. 10.3389/fpsyg.2018.01290 30090080 PMC6068388

[7] Appelbaum LG Schroeder JE Cain MS Mitroff SR. Improved visual cognition through stroboscopic training. Front Psychol. (2011) 2. 10.3389/fpsyg.2011.00276 22059078 PMC3203550

[8] Ballester R Huertas F Uji M Bennett SJ. Stroboscopic vision and sustained attention during coincidence-anticipation. Sci Rep. (2017) 7:17898. 10.1038/s41598-017-18092-5 29263340 PMC5738365

[9] Zwierko M Jedziniak W Popowczak M Rokita A. Effects of *in-situ* stroboscopic training on visual, visuomotor and reactive agility in youth volleyball players. PeerJ. (2023) 11:e15213. 10.7717/peerj.15213 37250711 PMC10211363

[10] Zwierko M Jedziniak W Popowczak M Rokita A. Effects of a 6-week stroboscopic training program on specific blocking reaction speed in young volleyball players. Phys Act Rev. (2024) 12:1–10. 10.16926/par.2024.12.16 PMC10905827 38424539

[11] Uzlaşır S Özdıraz KY Dağ O Tunay VB. The effects of stroboscopic balance training on cortical activities in athletes with chronic ankle instability. Phys Ther Sport. (2021) 50:50–8. 10.1016/j.ptsp.2021.03.014 33865218

[12] Yu C Cheng M An X Chueh T Wu J Wang K The effect of EEG neurofeedback training on sport performance: a systematic review and meta-analysis. Scand J Med Sci Sports. (2025) 35:e70055. 10.1111/sms.70055 40270441 PMC12019780

[13] Luo Y Cao Y Pan X Li S Koh D Shi Y. Effects of stroboscopic visual training on reaction time and movement accuracy in collegiate athletes: a systematic review and meta-analysis. Sci Rep. (2025) 15. 10.1038/s41598-025-10393-4 PMC12254360 40646137

[14] Das J Walker R Barry G Vitório R Stuart S Morris R. Stroboscopic visual training: the potential for clinical application in neurological populations. PLOS Digit Health. (2023) 2:e0000335. 10.1371/journal.pdig.0000335 37611053 PMC10446176

[15] Choi H Kim H You J Sung H. Effects of balance training with stroboscopic glasses on joint position sense, postural stability, and functional performance testing in chronic ankle instability national athletes: a single-blinded randomized controlled trial. J Mech Med Biol. (2024) 24:2440040. 10.1142/S0219519424400402

[16] Lee H Han S Page G Bruening DA Seeley MK Hopkins JT. Effects of balance training with stroboscopic glasses on postural control in chronic ankle instability patients. Scand J Med Sci Sports. (2022) 32:576–87. 10.1111/sms.14098 34775656

[17] Lee H Han S Hopkins JT. Visual disruption and neuromechanics during landing-cutting in individuals with chronic ankle instability. J Athl Train. (2024) 59:822–9. 10.4085/1062-6050-0379.23 38014796 PMC11340668

[18] Ellison P Jones C Sparks SA Murphy PN Page RM Carnegie E The effect of stroboscopic visual training on eye-hand coordination. Sport Sci Health. (2020) 16:401–10. 10.1007/s11332-019-00615-4

[19] Han S Lee H Son SJ Hopkins JT. The effects of visual feedback disruption on postural control with chronic ankle instability. J Sci Med Sport. (2022) 25:53–7. 10.1016/j.jsams.2021.07.014 34393051

[20] Miao Y Ge Y Wang D Mao D Song Q Wu R. Effects of visual disruption on static and dynamic postural control in people with and without chronic ankle instability. Front Bioeng Biotechnol. (2024) 12:1499684. 10.3389/fbioe.2024.1499684 39564099 PMC11574417

[21] Wilkins L Nelson C Tweddle S. Stroboscopic visual training: a pilot study with three elite youth football goalkeepers. J Cogn Enhanc. (2018) 2:3–11. 10.1007/s41465-017-0038-z

[22] Argilés M Maymó-Català L Navinés-Ferrer U Quevedo-Junyent L Erickson G. Effect of 1 h of training with action video game or stroboscopic goggles on visuocognitive skills related to sports performance. Sport Sci Health. (2025) 21:1773–81. 10.1007/s11332-025-01406-w

[23] Fortes LS Faro H Faubert J Freitas-Júnior CG Lima-Junior Dd Almeida SS. Repeated stroboscopic vision training improves anticipation skill without changing perceptual-cognitive skills in soccer players. Appl Neuropsychol Adult. (2025) 32:1123–37. 10.1080/23279095.2023.2243358 37552715

[24] Li T Wang X Wu Z Liang Y. The effect of stroboscopic vision training on the performance of elite curling athletes. Sci Rep. (2024) 14:31730. 10.1038/s41598-024-82685-0 39738499 PMC11685645

[25] Zwierko M Jedziniak W Popowczak M Rokita A. Effects of six-week stroboscopic training program on visuomotor reaction speed in goal-directed movements in young volleyball players: a study focusing on agility performance. BMC Sports Sci Med Rehabil. (2024) 16:59. 10.1186/s13102-024-00848-y 38424539 PMC10905827

[26] Kim K-M Estudillo-Martínez MD Castellote-Caballero Y Estepa-Gallego A Cruz-Díaz D. Short-term effects of balance training with stroboscopic vision for patients with chronic ankle instability: a single-blinded randomized controlled trial. Int J Environ Res Public Health. (2021) 18:5364. 10.3390/ijerph18105364 34069907 PMC8157596

[27] Palmer T Coutts AJ Fransen J. An exploratory study on the effect of a four-week stroboscopic vision training program on soccer dribbling performance. Braz J Mot Behav. (2022) 16:254–65. 10.20338/bjmb.v16i3.310

[28] Symeonidou E-R Ferris DP. Visual occlusions result in phase synchrony within multiple brain regions involved in sensory processing and balance control. IEEE Trans Neural Syst Rehabil Eng. (2023) 31:3772–80. 10.1109/TNSRE.2023.3317055 37725737 PMC10616968

[29] Hülsdünker T Gunasekara N Mierau A. Short- and long-term stroboscopic training effects on visuomotor performance in elite youth sports. Part 1: reaction and behavior. Med Sci Sports Exerc. (2021) 53:960–72. 10.1249/MSS.0000000000002541 33060548

[30] Zwierko T Tapia V Vera J Redondo B Morenas-Aguilar MD García-Ramos A. Enhancing reactive agility in soccer: the impact of stroboscopic eyewear during warm-up across fatigued and non-fatigued conditions. Eur J Sport Sci. (2024) 24:1798–808. 10.1002/ejsc.12224 39578413 PMC11621378

[31] Fransen J Lovell TWJ Bennett KJM Deprez D Deconinck FJA Lenoir M The influence of restricted visual feedback on dribbling performance in youth soccer players. Motor Control. (2017) 21:158–67. 10.1123/mc.2015-0059 27111662

[32] Beavan A Hanke L Spielmann J Skorski S Mayer J Meyer T The effect of stroboscopic vision on performance in a football specific assessment. Sci Med Footb. (2021) 5:317–22. 10.1080/24733938.2020.1862420 35077302

[33] Chen Y-C Tsai Y-Y Lin Y-T Hwang I-S. Enhancing anticipation control of the posture system in the elderly wearing stroboscopic glasses. J Neuroeng Rehabil. (2025) 22:104. 10.1186/s12984-025-01549-4 40325460 PMC12051271

[34] Harrison KD Dakin CJ Beethe AZ Louder T. Effects of stroboscopic vision on depth jump motor control: a biomechanical analysis. Bioengineering (Basel). (2024) 11:290. 10.3390/bioengineering11030290 38534564 PMC10967995

[35] Hülsdünker T Rentz C Ruhnow D Käsbauer H Strüder HK Mierau A. The effect of 4-week stroboscopic training on visual function and sport-specific visuomotor performance in top-level badminton players. Int J Sports Physiol Perform. (2019) 14:343–50. 10.1123/ijspp.2018-0302 30160560

[36] Kim K-M Kim J-S Grooms DR. Stroboscopic vision to induce sensory reweighting during postural control. J Sport Rehabil. (2017) 26. 10.1123/jsr.2017-0035 28605310

[37] Zhao S Mao D Sun W Song Q Zhang C Shen P Effects of clbp on physical stability of the elderly when crossing obstacles of different heights: 499. Med Sci Sports Exerc. (2021) 53:164–164. 10.1249/01.mss.0000760996.26280.af

[38] Talwar S. The effect of stroboscopic vision training on blind-folded straight-line walking. Int J Exerc Sci. (2024) 17. 10.70252/GZDG9193 38665163 PMC11042846

[39] Vasile AI Stănescu MI. Strobe training as a visual training method that improves performance in climbing. Front Sports Act Living. (2024) 6:1366448. 10.3389/fspor.2024.1366448 38832310 PMC11144897

[40] Guo Y Yuan T Peng J Deng L Chen C. Impact of sports vision training on visuomotor skills and shooting performance in elite skeet shooters. Front Hum Neurosci. (2024) 18:1476649. 10.3389/fnhum.2024.1476649 39606788 PMC11599212

