# Supplementary material for: Stroboscopic training effects on athletic performance and cognitive function across populations, purposes, and skill types: a systematic review and meta-analysis of randomized controlled trials
Source: Front Sports Act Living. 2025 Dec 11;7:1705693. doi: 10.3389/fspor.2025.1705693 (PMC12738379; doi:10.3389/fspor.2025.1705693)
Supplement: Supplementary file 1 [file Table1.docx]

| Study | Eligibility criteria | Random allocation | Concealed allocation | Similar baseline | Participant blinding | Investigator blinding | Assessor blindinge | Completeness of follow-up | Intention to treat | Between group comparisons | Point measures and variability | Total Score | Overall quality |
| --- | --- | --- | --- | --- | --- | --- | --- | --- | --- | --- | --- | --- | --- |
| Lee et al. 2024 | 1 | 1 | 0 | 1 | 0 | 0 | 0 | 1 | 1 | 1 | 1 | 7 | High quality ( low risk of bias ) |
| Choi et al. 2024 | 1 | 1 | 0 | 1 | 0 | 0 | 0 | 1 | 1 | 1 | 1 | 7 | High quality ( low risk of bias ) |
| Palmer et al. 2022 | 1 | 1 | 0 | 1 | 1 | 0 | 0 | 1 | 1 | 1 | 1 | 9 | High quality ( low risk of bias ) |
| Lee et al. 2021 | 1 | 1 | 0 | 1 | 0 | 0 | 1 | 1 | 0 | 1 | 1 | 7 | High quality ( low risk of bias ) |
| Kim et al. 2021 | 1 | 1 | 1 | 1 | 0 | 1 | 1 | 1 | 0 | 1 | 1 | 9 | High quality ( low risk of bias ) |
| Zwierko et al. 2024a | 1 | 1 | 0 | 1 | 0 | 0 | 1 | 1 | 1 | 1 | 1 | 8 | High quality ( low risk of bias ) |
| Zwierko et al. 2023 | 1 | 1 | 0 | 1 | 0 | 0 | 1 | 1 | 0 | 1 | 1 | 7 | High quality ( low risk of bias ) |
| Zwierko et al. 2024b | 1 | 1 | 0 | 1 | 0 | 0 | 1 | 1 | 1 | 1 | 1 | 8 | High quality ( low risk of bias ) |
| Zwierko et al. 2024c | 1 | 1 | 0 | 1 | 0 | 0 | 0 | 1 | 1 | 1 | 1 | 7 | High quality ( low risk of bias ) |
| Li et al. 2024 | 1 | 1 | 0 | 1 | 0 | 0 | 0 | 1 | 1 | 1 | 1 | 7 | High quality ( low risk of bias ) |
| Uzlaşır et al. 2021 | 1 | 1 | 0 | 1 | 0 | 0 | 0 | 1 | 1 | 1 | 1 | 7 | High quality ( low risk of bias ) |
| Argilés et al. 2025 | 1 | 1 | 0 | 1 | 0 | 0 | 0 | 1 | 1 | 1 | 1 | 7 | High quality ( low risk of bias ) |
| Ellison et al. 2020 | 1 | 1 | 0 | 1 | 0 | 0 | 0 | 1 | 1 | 1 | 1 | 7 | High quality ( low risk of bias ) |
| Fortes et al.2023 | 1 | 1 | 1 | 1 | 0 | 0 | 0 | 1 | 1 | 1 | 1 | 8 | High quality ( low risk of bias ) |
