# Supplementary figures and images for: Stroboscopic training effects on athletic performance and cognitive function across populations, purposes, and skill types: a systematic review and meta-analysis of randomized controlled trials
Source: Front Sports Act Living. 2025 Dec 11;7:1705693. doi: 10.3389/fspor.2025.1705693 (PMC12738379; doi:10.3389/fspor.2025.1705693)

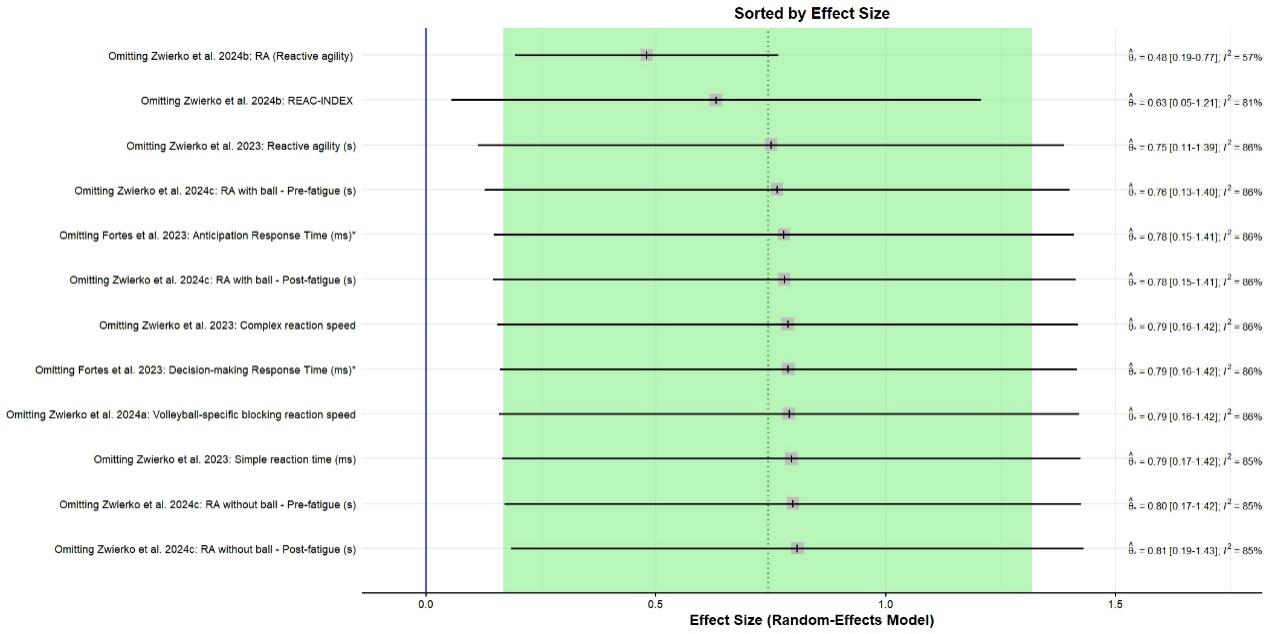


Figure A
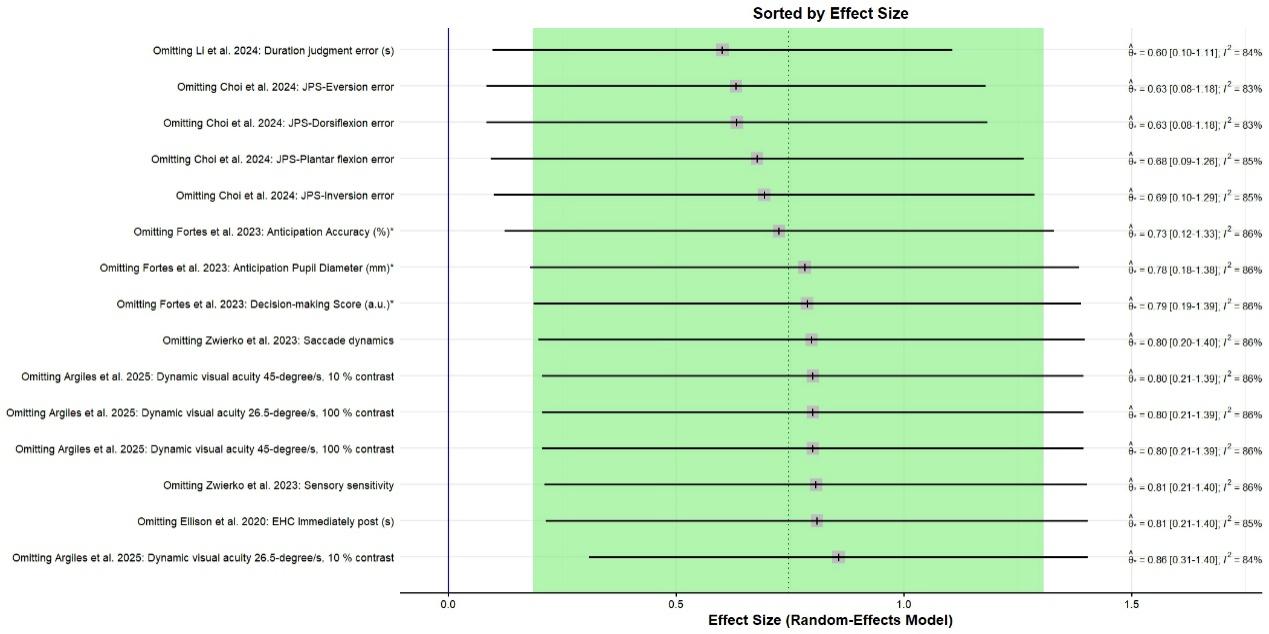


Figure B
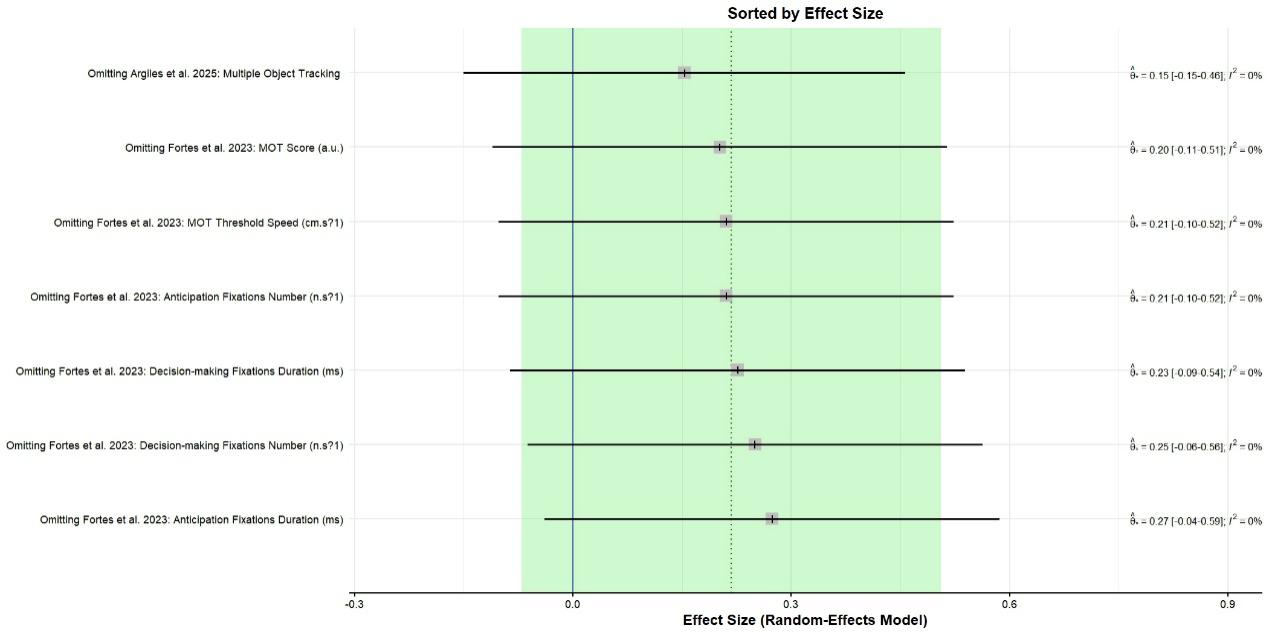


Figure C


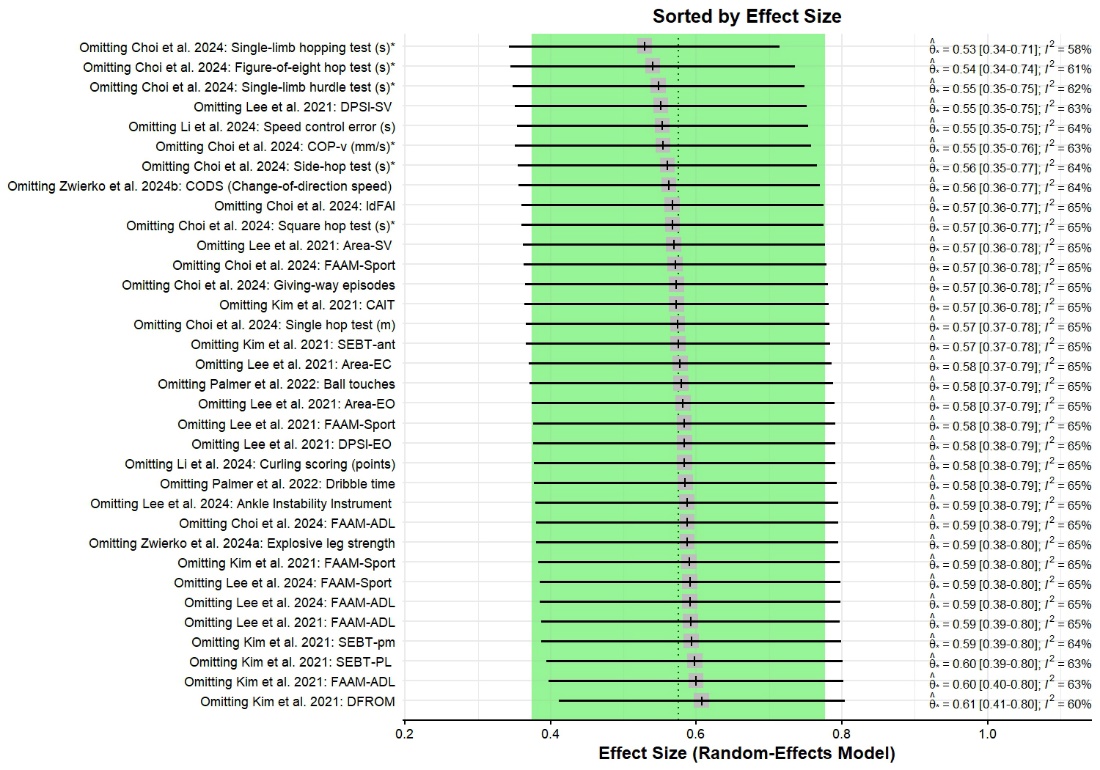


Figure D

Supplement: Supplementary file 3 [file Datasheet1.docx]
